# miR-1271 inhibits ERα expression and confers letrozole resistance in breast cancer

**DOI:** 10.18632/oncotarget.22359

**Published:** 2017-11-09

**Authors:** Tao Yu, Hai-Ru Yu, Jia-Yi Sun, Zhao Zhao, Shuang Li, Xin-Feng Zhang, Zhi-Xuan Liao, Ming-Ke Cui, Juan Li, Chan Li, Qiang Zhang

**Affiliations:** ^1^ Department of Breast Surgery, Cancer Hospital of China Medical University, Liaoning Cancer Hospital & Institute, Shenyang 110042, P.R.China; ^2^ Department of Medical Imaging, Cancer Hospital of China Medical University, Liaoning Cancer Hospital & Institute, Shenyang 110042, P.R.China

**Keywords:** estrogen receptor, miR-1271, letrozole, DDIT3, breast cancer

## Abstract

Attenuation of estrogen receptor α (ERα) expression via unknown mechanism(s) is a hallmark of endocrine-resistant breast cancer (BCa) progression. Here, we report that miR-1271 was significantly down-regulated in letrozole-resistant BCa tissues and in letrozole-resistant BCa cells. miR-1271 directly targeted the chromatin of DNA damage-inducible transcript 3 (DDIT3) gene. miR-1271 expression level was inversely correlated to *DDIT3* mRNA level in BCa biopsies. Form a mechanistic standpoint, reintroduction of exogenous miR-1271 could effectively restore ERα level via inhibiting DDIT3 expression, thereby potentiating letrozole sensitivity in BCa cells. Moreover, DDIT3 deregulation promoted letrozole-resistance by acting as a potent corepressor of *ESR1* transcription. Taken together, we have identified that disruption of the miR-1271/DDIT3/ERα cascade plays a causative role in the pathogenesis of letrozole resistance in BCa.

## INTRODUCTION

Estrogen receptor alpha (ERα) pathway plays an essential role in the development and progression of breast cancer (BCa). Current treatment for hormone-dependent postmenopausal BCa patients consists of two strategies to reduce the effects of estrogens on tumor growth: blocking estrogen binding to estrogen receptor (ER) with antiestrogens (*e.g.* tamoxifen) or inhibiting estrogen synthesis with aromatase inhibitors (AIs, *e.g.* exemestane, anastrozole and letrozole) [1]. Compared to antiestrogens, AIs block the conversion of androgens to estrogens and do not have agonist effects, so it can minimize the risk of endometrial cancer, stroke, or pulmonary embolism associated with tamoxifen [2]. Among different AIs, letrozole offers both symptomatic and survival benefits and remains the first-line option in endocrine therapy [3]. Despite the efficacy of letrozole treatments, a sizeable proportion (range 30% to 65%) of patients either does not respond to AIs or becomes resistant to them [4]. Oestrogen receptor 1 (*ESR1*; which encodes ERα) mutation in the region corresponding to the carboxy-terminal ligand-binding domain of the protein is an acquired AI-resistance mechanism. *ESR1* mutations are rare in treatment-naive primary BCa, but are frequently identified in metastatic disease samples, especially in studies of tumors progressing on serial endocrine therapy (including AIs resistance). Other potential mechanisms contributing to AI-resistance may consist of crosstalk between growth factor receptors (GFRs) signaling and ER, deregulated amplification of ESR1 and disruption of PI3K pathway in ER^+^ BCa, *et al* [5]. When resistance ultimately occurs, the disease becomes more difficult to control. Molecular profiling with massively parallel analyses has revealed that multiple mechanisms, including repression of ERα expression, activation of AKT and MAPK pathways, deregulation of microRNAs (miRNAs) and ERα-associated transcription factors, are certain to coexist to confer letrozole-resistant phenotypes [6]. In this scenario, continuous elucidation of the key molecules acting at different levels would help to enlarge our mechanistic understanding and provide valuable therapeutic clues.

miRNAs, a cluster of evolutionarily conserved, non-coding 22 nt RNAs, regulate gene expression post-transcriptionally by binding to the 3’untranslated region (3’UTR) of mRNAs to repress transcription or promote degradation. miRNA plays important regulatory roles under both physiological and pathological conditions [7]. In BCa, the modulation of endocrine resistance by miRNA is exemplified by, but not limited to, their involvement in regulating ERα. Let-7 miRNAs can induce tamoxifen sensitivity by down-regulation of ERα36. ERα36 is a 36-kD novel truncated isoform of ERα, and it transcribed from a promoter located in the first intron of the *ESR1* gene and lacks both transcriptional activation domains (AF-1 and AF-2), but retains the DNA-binding, dimerization and partial lig-and-binding domains [8]. Similarly, loss of miR-520 expression plays a promoting role in ERα-tumor progression via directly targeting NF-κB signaling [9]. Therefore, the regulation of ERα expression/stability by miRNAs in endocrine resistance of BCa can occur in both direct and indirect manner.

DNA damage-inducible transcript 3 (DDIT3), also known as CHOP10 or GADD154, is a member of the CCAAT/enhancer-binding protein family of transcription factors. As an apoptosis-related gene, DDIT3 is involved in various biological processes including regulating hepatocyte death, modulating the endoplasmic reticulum stress-mediated autophagy in colon cancer and regulating inflammatory response in Sjögren syndrome [10]. Of particular interest, up-regulation of DDIT3 can potentiate the combined treatment of clarithromycin and bortezomib [11], in BCa cells. Thus, These data collectively are indicative of a unique role of DDIT3 in BCa.

miR-1271 has been frequently identified as one of the most down-regulated tumor suppressor miRNAs in ovarian cancer [12], lung cancer [13] and BCa [14]. Besides, miR-1271 is a circulating miRNA [15, 16]. So further elucidation of its role in cancer biology should shed novel light on the development of a more effective method for non-invasive diagnosis and prognosis of cancer. In this study, we found a novel regulatory relationship between miR-1271 and DDIT3. We demonstrated that there is an inverse correlation between DDIT3 and miR-1271 in letrozole-resistant BCa tissues and in the letrozole-resistant cell line. Furthermore, DDIT3 deregulation promoted letrozole-resistance by acting as a potent corepressor of *ESR1* transcription. Overall, our systematic analysis will pave the way for a better understanding of the role of miR-1271/DDIT3/ERα cascade in BCa.

## RESULTS

### Down-regulation of miR-1271 in letrozole-resistant BCa cells

**Figure 1:**
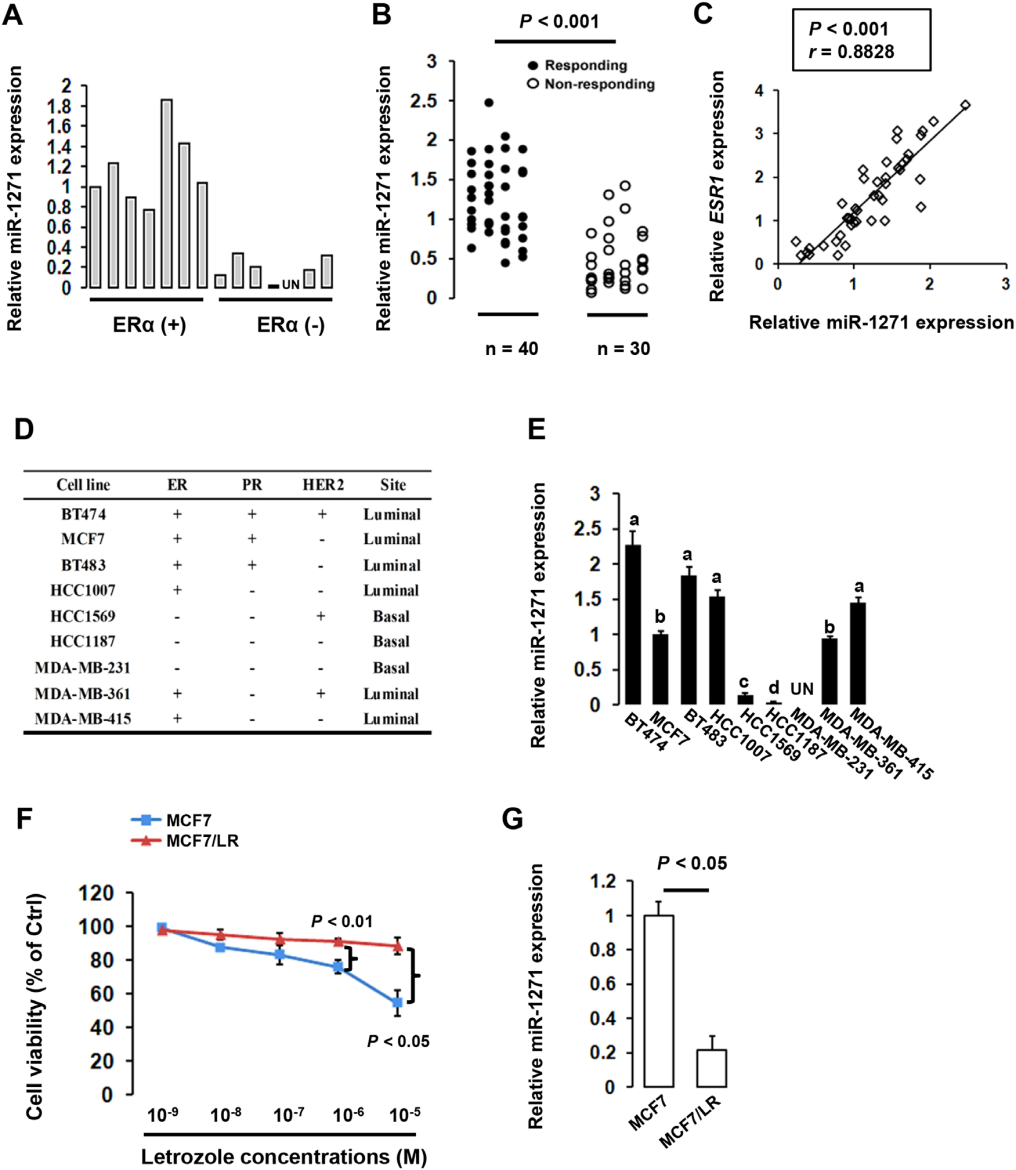
Repression of miR-1271 expression in letrozole-resistant breast cancer (BCa) **(A)** Expression of miR-1271 in BCa specimens from patients who had not received letrozole-based hormone therapy was determined using RT-qPCR. Relative expression levels of miR-1271 were obtained in each sample by normalization of the expressions of miR-1271 to that of the U6 snRNA signal. For presentation of data, expression levels of one patient from ER+ group were taken as 100% and the others were normalized accordingly, thus allowing semiquantitative comparison. **(B)** Comparison of miR-1271 expression in clinical samples of letrozole-responding (n=40) and non-responding (n=30) using RT-qPCR. **(C)** RT-qPCR analysis and subsequent *Pearson Chi-Square* test revealed that *ESR1* mRNA level was postively correlated with miR-1271 level in clinical BCa samples. **(D)** Genetic backgrounds of BCa cells used in the study. **(E)** RT-qPCR analysis of miR-1271 expression in different BCa cells. Relative expression levels of miR-1271 were obtained in each sample by normalization of the expressions of miR-1271 to that of the U6 snRNA signal. For presentation of data, expression levels of one patient from MCF7 cells were taken as 100% and the others were normalized accordingly, thus allowing semiquantitative comparison. **(F)** The letrozole-resistant MCF7 sublines (MCF7/LR) were developed as described in the ‘MATERIALS AND METHODS’ section. MCF7/LR cells (red line) and aromatase-overexpressing MCF7 cells (blue line) were seeded as described in ‘MATERIALS AND METHODS’. Three days later, cells were treated with steroid-free medium containing 25 nM Δ4A and different concentrations of letrozole. The medium was changed every 3 days, and the cells were counted 9 days later using the MTT assay. Viable cell numbers were finally determined and relative cell growth was calculated. **(G)** RT-qPCR analysis of miR-1271 level in MCF7 and MCF7/LR cells. Relative expression levels of miR-1271 were obtained in each sample by normalization of the expressions of miR-1271 to that of the U6 snRNA signal.

In the initial effort to explore the potential involvement of miR-1271 in BCa, we examined the miR-1271 level in 14 BCa tissue samples from patients who had not received letrozole-based hormone therapy (These newly-diagnosed BCa patients were neither in an adjuvant setting nor were metastatic cases). As shown in Figure 1A and Supplementary Figure 1, miR-1271 expression was remarkably reduced in ERα-negative BCa tissues compared with ERα-positive BCa tissues. To further investigate the correlation between miR-1271 deregulation and response to letrozole-based hormone therapy, we checked the miR-1271 expression in a total of 70 BCa patients who had received letrozole-based hormone monotherapy or letrozole-based hormone therapy combined with other chemotherapy [Among the 70 BCa patients, seven patients were previously enrolled in a Phase II clinical trial. To be specific, they received either pilaralisib tablets once daily (starting dose 200 mg) or voxtalisib capsules twice daily (starting dose 30 mg), each in combination with letrozole tablets 2.5 mg once daily administered 30 min after the pilaralisib dose or first (morning) voxtalisib dose]. The relative level of miR-1271 expression in non-responding tumor tissues (n=30) was significantly lower than that in responding tumor tissues (n=40) (*P*<0.001; Figure 1B). RT-qPCR analysis along with *Pearson Chi-Square* test revealed that *ESR1* mRNA level was positively correlated with miR-1271 level in clinical BCa samples (Figure 1C). Subsequent *in vitro* quantitative analysis showed that miR-1271 expression was notably down-regulated in ERα-negative BCa cells whereas PR or HER2 status had no effects on miR-1271 expression (Figure 1D and 1E, Supplementary Figure 2 and Supplementary Figure 3). To determine whether miR-1271 down-regulation is an acquired characteristic in BCa cells, we established a letrozole-resistant MCF7 subclone (MCF7/LR, Figure 1F, Supplementary Figure 4). The IC50 value of aromatase-overexpressing MCF7 cells (blue line in Figure 1E) was about 7.8 nmol/L, whereas the IC50 value of MCF7/LR was >1000 nmol/L. Interestingly, miR-1271 was found to be significantly decreased ∼3.6-fold in MCF7/LR cell line, compared with the parental MCF7 cells (Figure 1G). These data together suggest that down-regulation of miR-1271, which was tightly associated with ERα existence, might have potential roles in the development of letrozole resistance in BCa cells.

### Effects of manipulation of miR-1271 level on letrozole sensitivity

**Figure 2:**
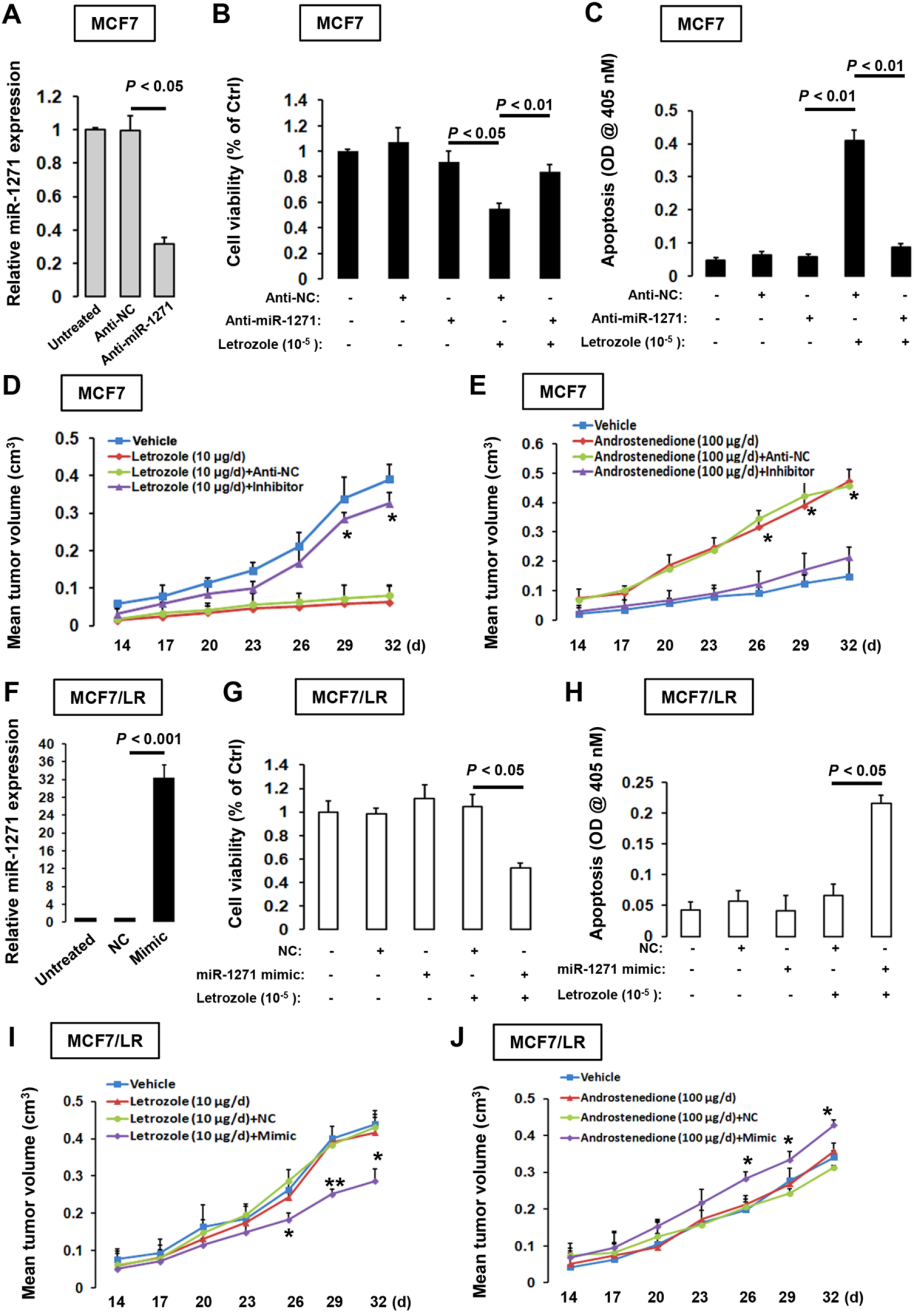
Manipulation of miR-1271 expression alters letrozole-sensitivity in BCa cells **(A)** RT-qPCR analysis of miR-1271 level in MCF7 cells transfected with anti-miR-1271 or empty control vector (Anti-NC). The untreated MCF7 cells, MCF7 cells transfected with anti-NC and MCF7 cell transfected with anti-miR-1271 in (A) were treated with 10^-5^ M of letrozole for 9 days, followed by MTT assay **(B)** and apoptotic ELISA **(C)**. **(D)** Each mouse received s.c. injections at one site on each flank with 0.1 mL of suspension of transfected MCF7 cells (2×10^7^ cells/mL). Mice were then injected s.c. daily for 32 days with vehicle, androstenedione (Δ4A, 100 μg/d), or androstenedione plus letrozole (10 μg/d) from the day of inoculation. Tumor volumes were measured every 3 days. **(E)** Tumor xenografts experiment was carried out as described in (D) except that after inoculation mice were treated with vehicle or androstenedione. **(F)** RT-qPCR analysis of miR-1271 level in MCF7/LR cells transfected with miR-1271 mimic or empty control vector (NC). Transfected MCF7/LR cells in (F) were treated with 10^-5^ M of letrozole for 9 days, followed by MTT assay **(G)** and apoptotic ELISA **(H)**. **(I)** Each mouse received s.c. injections at one site on each flank with 0.1 mL of suspension of transfected MCF7/LR cells (2×10^7^ cells/mL). Mice were then injected s.c. daily for 32 days with vehicle, androstenedione (Δ4A, 100 μg/d), or androstenedione plus letrozole (10 μg/d) from the day of inoculation. Tumor volumes were measured every 3 days. **(J)** Tumor xenografts experiment was carried out as described in (H) except that after inoculation mice were treated with vehicle or androstenedione.

To investigate the role of endogenous miR-1271 in BCa cells, we knocked down miR-1271 expression using its inhibitors (Figure 2A). Suppression of miR-1271 expression in MCF7 cells significantly reduced letrozole-elicited apoptosis and thereby restored cell viability to the normal level in the presence of letrozole treatment (Figure 2B and 2C). Furthermore, when transfected MCF7 cells were inoculated into ovariectomized nude mice injected subcutaneously daily with letrozole (10μg/d) along with androstenedione (Δ4A, 100μg/d), tumors grew much larger in the cells transfected with miR-1271 inhibitors. Consistently, when MCF7 cells were depleted with miR-1271 expression by inhibitors, tumors grew equally well in the presence and absence of androstenedione (Figure 2D and 2E, Supplementary Figure 5). These findings provide *in vivo* evidence that endogenous miR-1271 is required for letrozole sensitivity in BCa cells. To ask whether miR-1271 has an causative effect on letrozole sensitivity, we transfected MCF7/LR cells with miR-1271 mimic or negative controls (Figure 2F). When exposed to letrozole treatment, the cell proliferation was reduced whereas apoptosis was notably stimulated in MCF7/LR-mimic cells, compared to those in MCF7/LR-NC cells (Figure 2G and 2H). In line with the *in vitro* observations, MCF7/LR-mimic cells-derived tumors grew much smaller in the presence of daily injection with androstenedione and letrozole (Figure 2I). Moreover, MCF7/LR-mimic cells-derived tumors grew much larger upon androstenedione injection, when compared to MCF7/LR-NC cells-derived tumors (Figure 2J). Thus, up-regulation of miR-1271 could enhance the letrozole sensitivity in MCF7/LR cells.

### Association between miR-1271 and ERα existence

**Figure 3:**
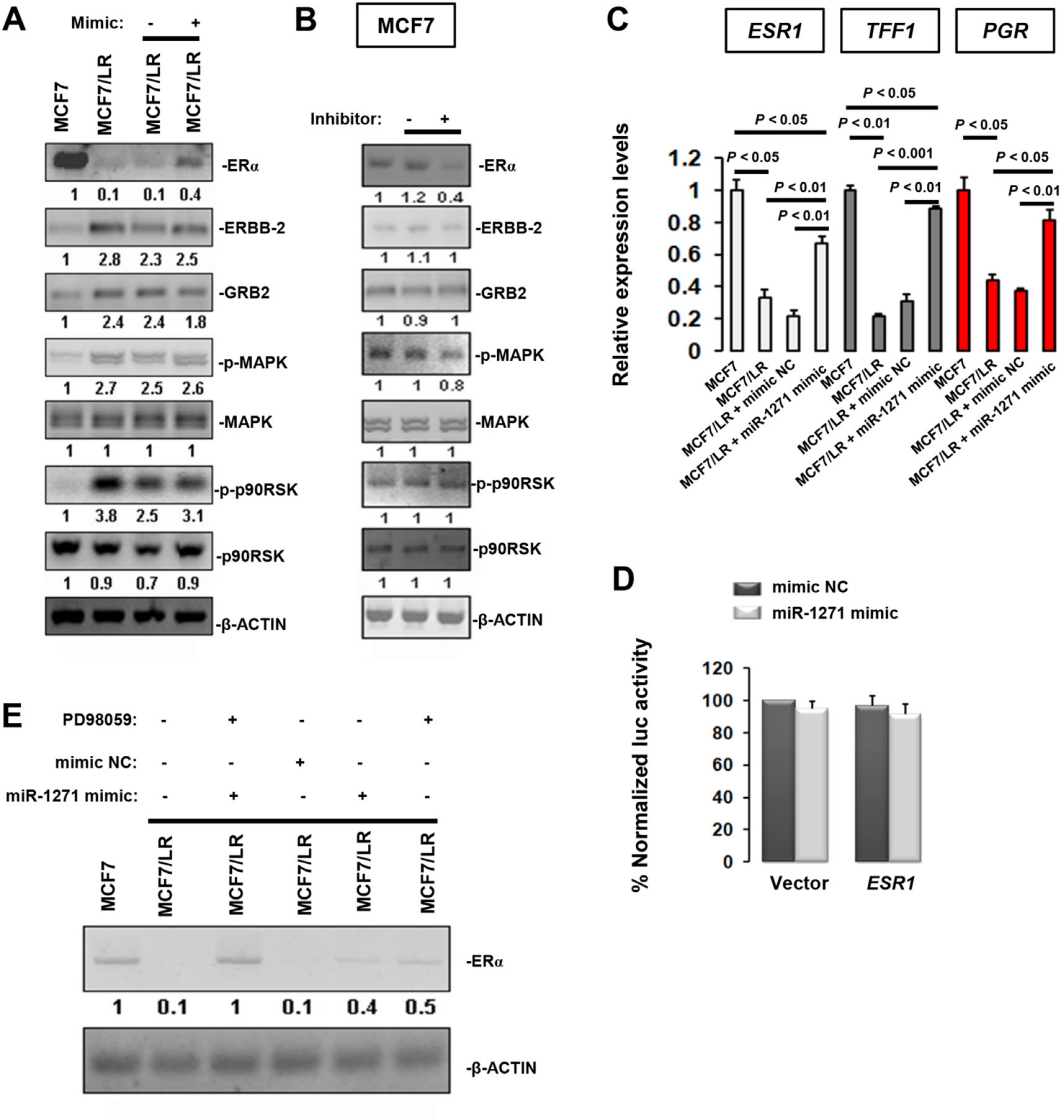
Indirectly modulation of ERα expression by miR-1271 **(A)** Immunoblotting analysis of ERα, growth factor receptor ERBB-2, and adapter proteins GRB2 and MAPK pathway in BCa cells. Relative expression levels of target proteins were obtained in each sample by normalization of the expressions of specific targets to that of the β-ACTIN signal. For presentation of data, expression levels of MCF7 were taken as 100% and the others were normalized accordingly, thus allowing semiquantitative comparison. Numbers below the blots represent fold change in protein expression compared with the MCF7 control obtained by densitometric analysis. **(B)** 48 h after transfection, the untreated MCF7 cells, MCF7 cells transfected with anti-NC and MCF7 cell transfected with anti-miR-1271 cells were subjected to immunoblotting analysis of different targets. **(C)** 48 h after transfection, untreated MCF7 cells, untreated MCF7/LR cells, MCF7/LR cells transfected with mimic NC and MCF7/LR cells transfected with miR-1271 mimics were subjected to RT-qPCR analysis of different targets. **(D)** miR-1271 does not target the *ESR1* 3’UTR in a luciferase reporter assay. **(E)** Immunoblotting analysis of ERα level in MCF7/LR cells treated with miR-1271 mimic, NC or MAPK pathway inhibitor PD98059.

To determine the potential mechanism(s) underlying our observations, immunoblotting analysis was done in MCF7 and MCF7/LR cells. In MCF7/LR cells, ERα expression was dramatically decreased, while growth factor receptor ERBB-2, adapter proteins GRB2 and MAPK signaling proteins p-MAPK and downstream effector of MAPK (p-p90RSK) were all up-regulated. Of note, transfection with miR-1271 mimic in MCF7/LR cells could partially restore ERα level but had no effects on other proteins (Figure 3A). To further confirm the modulation of ERα expression by miR-1271, we examined the MCF7 cells transfected with miR-1271 inhibitor. Suppression of miR-1271 caused a ∼60% reduction in ERα level but exerted no effects on other proteins expression (Figure 3B). To determine whether miR-1271 affects ERα pathway, we assessed the expression levels of ERα-target genes (*TFF1* and *PGR*) using RT-qPCR. Expression levels of *ESR1*, *TFF1* and *PGR* were all substantially down-regulated in MCF7/LR cells, whereas transfection with miR-1271 mimic could partially restore *ESR1* and *TFF1* levels, and fully recover *PGR* expression (Figure 3C). To ask whether miR-1271 directly targets *ESR1*, we co-transfected COS-1 cells with pLightSwitch-Luc-*ESR1* and miR-1271 mimic or mimic-NC. The luc activity was found to be unaltered in the presence or absence of miR-1271 overexpression (Figure 3D). It was previously shown that inhibitors of the MAPK cascade is useful in blocking pathways activated by long-term letrozole treatment [17]. Consistently in our study, treatment with miR-1271 mimic or MAPK inhibitor (PD98059) both effectively but partially restored ERα levels in MCF7/LR cells (Figure 3E). Moreover, PD98059 alone could increase *ESR1* transcript expression in MCF7/LR cells (Supplementary Figure 6A) and the combined treatment with miR-1271 mimic + PD98059 exhibited most effective rescuing effect on MCF7/LR cells and could almost recover the *ESR1* transcript level to that in MCF7 cells (Supplementary Figure 6B). Additionally, the rescuing effect of combined treatment with miR-1271 mimic + PD98059 was exerted in a time-dependent manner (Supplementary Figure 6C). Thus, in cooperation with MAPK pathway, miR-1271 may modulate ERα expression in an indirect manner.

### MiR-1271 directly targets DDIT3

**Figure 4:**
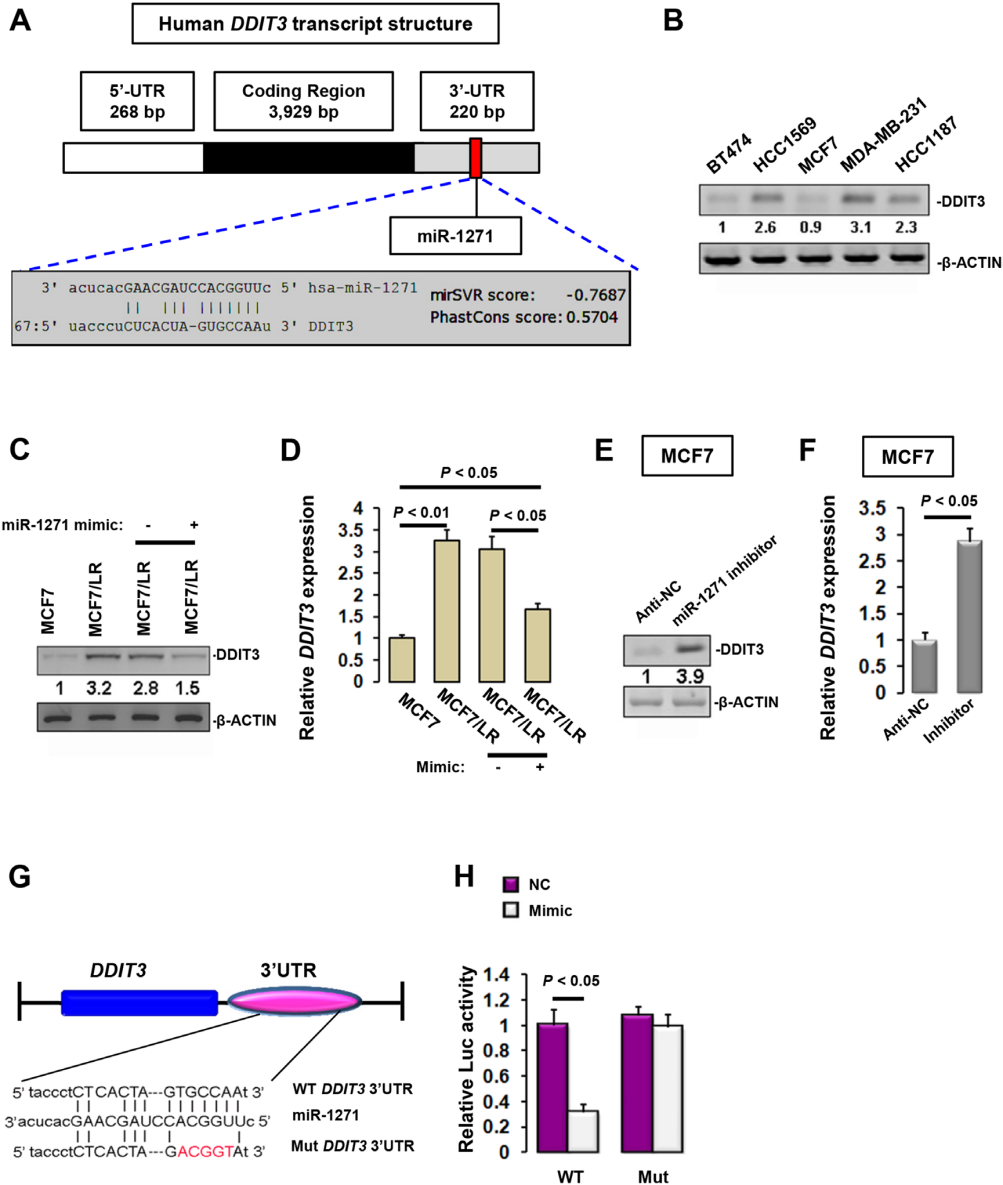
miR-1271 directly targets DDIT3 **(A)** Prediction of DDIT3-targeting sites of miR-1271 using miRanda. **(B)** Immunoblotting analysis of DDIT3 level in various BCa cells. 48 h after transfection, ostensible MCF7 cells, untreated MCF7/LR cells, MCF7/LR cells with mimic NC transfection, and MCF7/LR cells with miR-1271 mimic transfection were subjected to immunoblotting **(C)** or RT-qPCR **(D)** analyses of DDIT3 level. 48 h after transfection with miR-1271 inhibitor or Anti-NC, MCF7 cells were subjected to immunoblotting **(E)** or RT-qPCR **(F)** analyses of DDIT3 level. **(G)** schematic diagram showing the mutation of miR-1271 binding site in the *DDIT3* 3’UTR. **(H)** Relative luciferase activity was analyzed after wild-type or mutant 3’UTR reporter plasmids were co-transfected with different plasmids in COS-1 cells.

To further elucidate the mechanism(s) underlying miR-1271 function, we searched two miRNA target prediction databases (Targetscan and microRNA.org), which showed that DDIT3 might be one of the potential targets (Figure 4A). In good contrast to miR-1271 expression profile, DDIT3 expression was substantially robust in ERα-negative HCC1569, MDA-MB-231 and HCC1187 cells (Figure 4B). Consistently, DDIT3 expression in MCF7/LR cells was much higher than that in MCF7 cells, and this stimulated DDIT3 expression was efficiently but partially reversed when MCF7/LR cells was transfected with miR-1271 mimic (Figure 4C and 4D). To ask whether enhanced DDIT3 expression is causative of or a result of miR-1271 deficiency, we assessed DDIT3 level in MCF7 cells transfected with miR-1271 inhibitor or Anti-NC. Repression of endogenous miR-1271 significantly evoked DDIT3 up-regulation at both transcriptional and translational levels (Figure 4E and 4F). To confirm whether miR-1271 directly targets *DDIT3*, we co-transfected COS-1 cells with pLightSwitch-Luc-*DDIT3*/3’UTR and miR-1271 mimic or mimic-NC. 48 h later, the luc activity was determined to be significantly inhibited by about 68.4% in cells co-transfected with pLightSwitch-Luc-*DDIT3*/3’UTR and miR-1271 mimic, whereas mutation of the 3’UTR binding site effectively abolished miR-1271 mimic-mediated inhibition of Luc-*DDIT3*/3’UTR activity (Figure 4G and 4H). Moreover, by using *Pearson Chi-Square* test, it was observed that the miR-1271 expression level was inversely correlated to *DDIT3* mRNA level in BCa biopsies (Supplementary Figure 7). Therefore, miR-1271 may negatively regulate *DDIT3* expression by targeting its 3’UTR.

### Repression of ESR1 transcription by DDIT3

**Figure 5:**
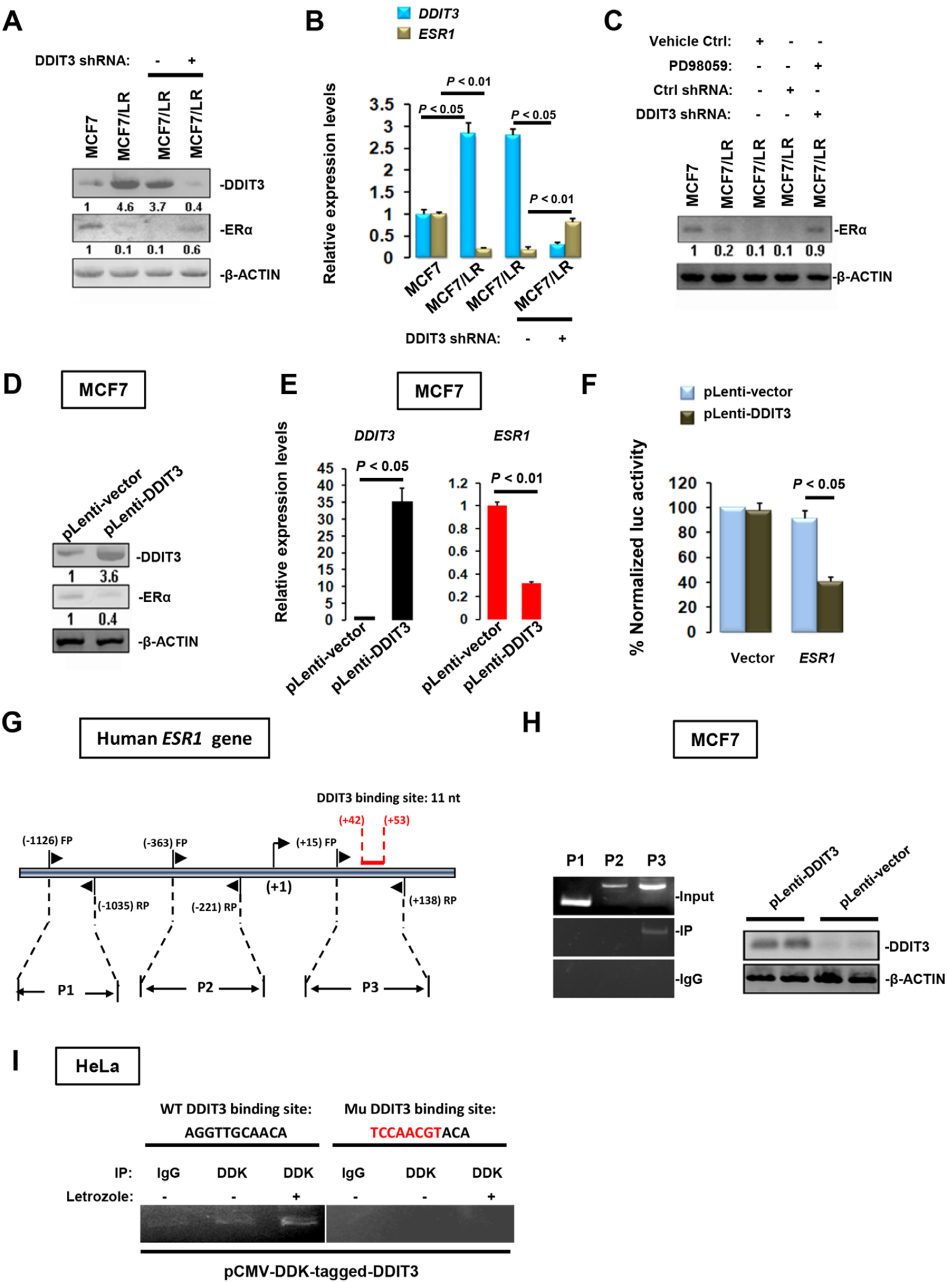
Transcriptional repression of *ESR1* by DDIT3 Immunoblotting **(A)** and RT-qPCR **(B)** analyses of DDIT3 and ERα levels in BCa cells treated with DDIT3 shRNA or negative control. **(C)** Effect of combined treatment with DDIT3 shRNA and PD98059 on ERα levels in MCF7/LR cells. 48 h after transfection with pLenti-DDIT3 or empty vector, MCF7 cells were subjected to immunoblotting **(D)** or RT-qPCR **(E)** analyses of DDIT3 and ERα expression. **(F)** DDIT3 directly targets the *ESR1* promoter in a luciferase reporter assay. **(G)** Simplified structure of the potential binding site of DDIT3 onto *ESR1* promoter. **(H)** ChIP analysis showing recruitment of DDIT3 onto a specific region of the *ESR1* promoter in MCF7 cells. **(I)** ChIP analysis showing that letrozole treatment could enhance the recruitment of DDIT3 onto *ESR1* promoter and mutation of DDIT3 binding sites in the *ESR1* promoter region substantially abolished the recruitment.

To explore the DDIT3 function in letrozole resistance, we examined its expression in different cell models. The expression of DDIT3 in MCF7/LR cells was significantly higher than that in MCF7 cells. Interestingly, inhibition of DDIT3 expression by shRNA could rescue the ERα expression in MCF7/LR cells at both translational and transcriptional levels (Figure 5A and 5B). Moreover, treatment with DDIT3 shRNA together with PD98059 could restore ERα expression (Figure 5C) or *ESR1* transcripts levels (Supplementary Figure 8) to the normal level in MCF7/LR cells. To ask whether enhanced DDIT3 expression has a causative role in ERα suppression, we stably transfected MCF7 cells with pLenti-GIII-CMV-DDIT3. DDIT3 overexpression notably inhibited ERα expression at both protein and mRNA levels (Figure 5D and 5E). Additionally, DDIT3 efficiently repressed the *ESR1* transcriptional activities in COS-1 cells as measured by promoter reporter assay (Figure 5F). By using QIAGEN Website, we have identified a potential DDIT3 binding site on *ESR1* promoter. To verify this, we performed ChIP assay, using primers encompassing different fragments of the *ESR1* promoter region (Figure 5G). DDIT3 was recruited to the +15 to +138 bp region of the *ESR1* promoter (Figure 5H). To further confirm the binding site of DDIT3 in *ESR1* promoter, we cloned the *ESR1* promoter into pCI Mammalian Expression Vector and simultaneously mutated the binding sites using a commercial Site-Directed Mutagenesis Kit. Subsequent ChIP assay in HeLa cells revealed that the recruitment of DDIT3 onto *ESR1* promoter could be augmented by letrozole treatment. Furthermore, mutation of binding sites in *ESR1* promoter region completely abolished the recruitment of DDIT3 onto *ESR1* promoter (Figure 5I), confirming that DDIT3 directly targets *ESR1* gene chromatin.

### DDIT3 deregulation is responsible for miR-1271-deficiency induced letrozole-resistance

**Figure 6:**
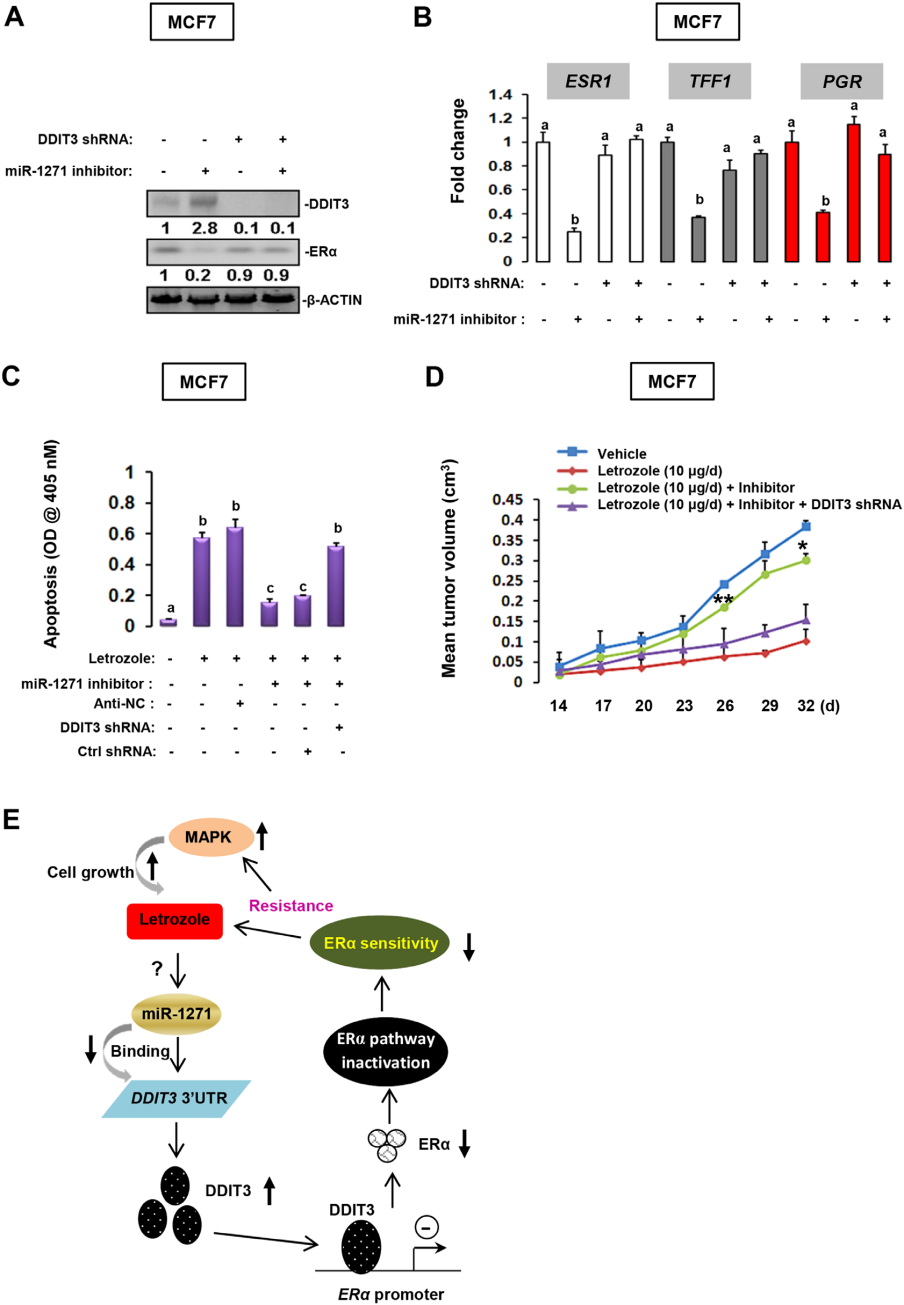
DDIT3 is the main downstream effector of miR-1271 signaling **(A)** Effect of DDIT3 knockdown on miR-1271 deficiency-impaired ERα expression was evaluated using immunoblotting. **(B)** Effect of DDIT3 knockdown on miR-1271 deficiency-impaired ERα pathway was evaluated using RT-qPCR. Different superscript letters denote groups that are statistically different (*P*<0.05). **(C)** Effect of DDIT3 knockdown on miR-1271 deficiency-impaired letrozole-sensitivity was determined using apoptotic ELISA. **(D)** We established the MCF7 cells stably deficient of DDIT3 expression according to a previously reported protocol. Subsequently, cells were subjected to tumor xenografts experiment to reveal the effect of DDIT3 knock-down on tumor growth *in vivo*. **(E)** Working model in the current study.

Finally, we determined whether DDIT3 is the main downstream effector of miR-1271 signaling. Treatment with miR-1271 inhibitor significantly evoked DDIT3 expression and impaired ERα expression. In contrast, treatment with DDIT3 shRNA could effectively abolish miR-1271 inhibitor-induced DDIT3 upregulation and thereby restored ERα level (Figure 6A). The latter observation was confirmed by examination of *TFF1* and *PGR* transcription using RT-qPCR (Figure 6B). From a functional standpoint, repression of DDIT3 expression by shRNA could fully restore miR-1271-deficiency impaired letrozole-sensitivity in MCF7 cells (Figure 6C). To further provide the *in vivo* evidence for the involvement of DDIT3 in miR-1271 function, we established the MCF7 cells stably deficient of DDIT3 expression (Supplementary Figure 9) according to a previously reported protocol [18]. As expected, DDIT3 suppression effectively rescued the miR-1271-deficiency impaired letrozole-sensitivity (Figure 6D), and stimulated ERα expression in MCF7 cells (Supplementary Figure 9). Additionally, we also confirmed the reciprocal regulation of the miR-1271/DDIT3/ERα cascade in another luminal BCa cell line (BT483) (Supplementary Figure 10). Taken together, that the available data suggest that DDIT3 may be a down-stream effector of miR-1271 signaling in letrozole-resistant MCF7 cells.

## DISCUSSION

Deciphering miRNAs deregulation associated with acquired AIs resistance could provide important new clues to improve this first-line therapy. Here, we found that miR-1271 was significantly down-regulated in letrozole-resistant BCa tissues, compared to letrozole-sensitive BCa tissues. Functionally, restoration of miR-1271 potentiated the letrozole-sensitivity in letrozole-resistant MCF7/LR cells, whereas inhibition of miR-1271 exerted an opposite effect in letrozole-sensitive MCF7 cells, suggesting that miR-1271 may be a tumor suppressor during BCa progression. In favor of our assumption, miR-1271 has been reported to inhibit migration, invasion and epithelial-mesenchymal transition in pancreatic cancer cells [19]. Likewise, miR-1271 could inhibit cell proliferation in ovarian cancer [12], non-small cell lung cancer [20] and multiple myeloma [21]. Thus, the tumor suppressive role appears to be relatively conserved in miR-1271.

In related studies, letrozole-resistant tumor growth occurs by activating alternate signaling pathways (such as MAPK and AKT pathways). Activated MAPK can regulate targets in the cytosol (p90RSK) and thereby activate ERα, either directly by phosphorylation at Ser^118^, or indirectly through p90RSK by phosphorylation at Ser^167^ and these phosphorylations activate ERα transcriptional activity in the absence of estrogens as would be seen in AI-treated patients with ERα+ primary tumors [22, 23]. Thus, deregulation of MAPK cascade has been proposed to be an important contingent effects along with the decrease of ERα expression in letrozole-resistant BCa [17]. However, there are few reports explaining how ERα expression is disrupted in letrozole-resistance. Recent advance has revealed that overexpression of miR-125b promotes resistance to letrozole by activating AKT/mTOR pathway [24]. Actually, decreased ERα expression caused by miRNAs deregulation has been frequently described in tamoxifen-resistant BCa progression: miR-221/222, miR-342-3p and miR-873 can all down-regulate ERα protein expression [9]. In our case, transfection with miR-1271 mimic could partially rescue ERα protein level in MCF7/LR cells, as effectively as treatment with MAPK pathway inhibitor PD98059, suggesting that miR-1271 has a positive modulatory effect on ERα expression. Of note, this stimulatory effect of miR-1271 appeared to be exerted in an indirect manner, because we could not find any binding site of miR-1271 on 3’UTR of *ESR1* chromatin and co-transfection with pLightSwitch-Luc-*ESR1* and miR-1271 mimic had no effect on luc activity. Nevertheless, potentiation of ERα sensitivity by regulating miRNA-modulated ERα level and blocking MAPK cascade may lead to most effective inhibition of growth of ER-positive BCa cells, and should be considered as a combined strategy in future therapeutic study and application.

ERα expression and activity is tightly controlled by many transcription factors, *e.g.*, AP-1, Sp-1 and NF-κB [9]. Our findings extend these understanding by identifying DDIT3 as a potent corepressor of *ESR1* transcription. DDIT3 recruitment to the *ESR1* promoter after letrozole treatment was even more robust than that without letrozole treatment, reemphasizing the essential regulatory role of DDIT3 in DNA damage. Additionally, the transcriptional repression may represent an intrinsic idiosyncracy of DDIT3. DDIT3 represses *MyoD* transcription to delay myoblast differentiation [25]. Similarly in multipotent mesenchymal cells, DDIT3 selectively represses osteoblastic and chondrocytic transcription [26]. However, the upstream mechanisms controlling DDIT3 expression under different pathologies are poorly characterized. To be noted, many transcription factors associated with ERα expression have been found to be regulated by miRNA. For instance, histone deacetylase (HDAC) complexes associated with the metastatic-associated protein 1 (MTA1) corepressor mediate ERα transcriptional repression by heregulin-beta1 [27] whereas MTA1 is a direct target of miR-421 in BCa [28]. Likewise, ERα coactivator SRC-3 overexpression results in constitutive activation of ERα-mediated transcription, BCa progression, and resistance to tamoxifen [29]. SRC-3 translation is repressed by miR-17-5p and miR-17-5p overexpression inhibits E2-induced proliferation [9]. We believe that DDIT3 is also such a striking example because mutation of the 3’UTR binding site effectively abolished miR-1271 mimic-mediated inhibition of Luc-*DDIT3*/3’UTR activity, and treatment with DDIT3 shRNA enhanced ERα expression, and could fully restore ERα level even in the presence of miR-1271 inhibitor. Taken together, distinct miRNA and ERα interactors may integrate into a complicated signal network to modulate ERα activity during the BCa progression, which warrants further investigation.

DDIT3 is often portrayed as a key initiating factor of endoplasmic reticulum stress-related cell death [10]. In our study, however, inhibition of DDIT3 expression by shRNA effectively restored ERα expression and reversed miR-1271-deficiency impaired letrozole-sensitivity in MCF7 cells, suggestive of an opposite tumor promoting effect. This discrepancy may reflect the complexity of DDIT3 function. It has been shown that the promoter of DDIT3 gene contains several binding sites for activator protein-1 (AP-1) [30]. AP-1 transcription factors are known to participate in both the induction and prevention of apoptosis, and the exact outcome is highly tissue- and developmental-stage-specific [31]. So it is likely that the cellular context is critical for determining the contribution of DDIT3 to cellular fates, and the role of DDIT3 in cancerous progression should be considered within the context of a complex network of DDIT3-associated transcriptional factors that respond simultaneously to a wide range of signal transduction pathways.

From a clinical standpoint, measurement of miR-1271 expression in letrozole-resistant BCa biopsies holds great promise, as miRNA can now be extracted not only from frozen tumor samples but also from formalin-fixed, paraffin-embedded samples as well as from plasma [9]. In this regard, testing patients for miR-1271/DDIT3 expression ratios, along with ERα level, may provide more accurate prognostic information and could influence the recommended course of letrozole treatment.

To be noted, data from the current study should be interpreted with caution as clinical resistance to hormone therapy can be observed even in the patients in whom the expression of ERα is preserved or unaltered. To this end, the “non-responding” patients shown in Figure 1B must have had ERα-positive BCa, because they received adjuvant hormone therapy. Three possibilities may account for these discrepancies regarding the effect of miR-1271/DDIT3/ERα cascade on BCa cells: (1) Molecular profiling analyses has frequently revealed an unexpectedly high level of heterogeneity in BCa. Therefore, it is very likely that the effect of miR-1271/DDIT3/ERα cascade may only exist in partial cases of ERα-positive BCa. (2) Those hormone-resistant patients with normal ERα expression level may have harbor certain *ESR1* mutations affecting interaction with miR-1271/DDIT3 signaling. (3) miR-1271 may also directly target the downstream genes of ERα pathway without affecting total ERα expression level. The last two interesting possibilities are presently under evaluation in our laboratory.

## CONCLUSIONS

In summary, our work demonstrates that down-regulation of miR-1271 is associated with letrozole resistance in BCa, and DDIT3 is a direct target of miR-1271. DDIT3 up-regulation caused by miR-1271-deficiency attenuates letrozole sensitivity in BCa cells via its transcriptional regulation property. A major pathway in this response is the direct negative control of *ESR1* transcription, which in turn governs the ability of miR-1271/DDIT3 cascade to modulate endocrine therapy-induced apoptosis in BCa cells (Figure 6E).

## MATERIALS AND METHODS

### Human samples

Female patients with primary BCa and known clinical follow-up who had not received any therapy before surgery and who relapsed, or not, while receiving endocrine therapy and/or chemotherapy were recruited from Department of Breast Surgery in Liaoning Cancer Hospital & Institute during May 2010 and December 2013. Primary BCa tissue samples, obtained from radical mastectomy specimens, were divided into ‘‘Responding’’ (complete or partial response) and ‘‘Non-responding’’ (stable or progressive disease) groups according to medical image analysis and detection of serum tumor markers within 1 year after drug withdrawal. The clinicopathologic characteristics of the patients are summarized in Supplementary Table 1. Patients was subdivided into BCa subtypes according to the *St Gallen* description [7]. Informed consent was obtained from all patients, and the study, conformed to the standards set by the 2008 Revised *Declaration of Helsinki*, was approved by the hospital’s ethics committee.

### Cell treatment

Cells used in the current study were all obtained from American type culture collection (ATCC; Rockville, MD, USA). All the cells were recently authenticated in December 2015 by the short tandem repeat analysis method using Promega Power-Plex1.2 analysis system (Genewiz Inc, Suzhou, China). Cells were cultured in DMEM supplemented with 10% fetal bovine serum, 1% penicillin/streptomycin at 37°C in 5% CO_2_ and 95% humidified air. The letrozole-resistant BCa cells, denoted MCF7/LR, was established as described elsewhere [17]. Briefly, after transfection with the human aromatase *CYP19A1* gene (SinoBiological, Beijing, China) and subsequent Hygromycin selection (100μg/ml, Takara, Dalian, China), stably transfected MCF7 cells were inoculated at the flanks with 0.1 ml of cell suspension (2×10^7^ cells/ml) in ovariectomized female nude mice. Stable transfection of aromatase provides an endogenous nonovarian source of estrogen to stimulate tumor growth derived from MCF7 cells inoculation [32]. 4 weeks after inoculation, mice were injected with vehicle (control), or letrozole (10μg/d, Sigma-Aldrich, Shanghai, China), along with the aromatase substrate androstenedione (Δ4A, 100μg/d, Sigma-Aldrich), for the duration of the experiment. At the end of 56 weeks of letrozole injection, MCF7/LR cells were isolated and maintained in phenol red-free IMEM supplemented with 5% charcoal/dextran-treated FBS, 1% penicillin/streptomycin, 100 μg/ml Hygromycin, and 1 μmol/l of letrozole. Animal work was approved by the local ethics committee.

To alter the expression levels of miR-1271, cells were incubated with miR-1271 mimics, miR-1271 inhibitors (anti-miR-1271), or corresponding controls at a concentration of 50 nM (GenePharma, Shanghai, China) in 100 μl culture medium without serum and antibiotics for 48 h, using Lipofectamine 2000 (Thermo Fisher Scientific, Waltham, MA, USA). To knockdown the endogenous DDIT3, cells were transiently transfected with pAV-U6-GFP-DDIT3 or vectors (Vigene, Rockville, MD, USA). To stably knockdown DDIT3 expression, MCF7 cells were transfected with DDIT3 shRNA (GE Dharmacon, Lafayette, CO, USA), followed by 0.1 μg/ml puromycin (Sigma-Aldrich) selection [18]. To overexpress the exogenous DDIT3, MCF7 cells were transfected with pLenti-GIII-CMV-DDIT3 or vectors, followed by 0.1 μg/ml puromycin selection according to the manufacturer’s instructions. In some cases, cells were pretreated for 2 h with the MAPK pathway inhibitor PD98059 (10 μM; Sigma).

### Cell survival assay

Cells were plated in 24-well plates at a density of 1×10^4^/ml in 100 ml of steroid-free medium per well. Three days later, cells were treated with steroid-free medium containing 25 nM Δ4A and different concentrations of letrozole. The medium was changed every 3 days, and the cells were counted 9 days later using the MTT assay (Millipore, Billerica, MA, USA). Final absorbance was measured in triplicate with a microplate reader at 570 nM (Bio-Rad680). The results were expressed as a percentage of the cell number compared with the vehicle-treated wells (control).

### Evaluation of apoptosis

Cells were treated with Δ4A (25 nM) and letrozole (10^-5^mol/l) for 9 days, followed by the apoptotic assay using an apoptosis ELISA kit (Roche Diagnostics, Mannheim, Germany) [33]. The final spectrophotometry was developed using peroxidase substrate and the absorbance was measured in triplicate at 405 nM.

### Tumor xenograft

Cells were washed by PBS and resuspended in Matrigel (10 mg/mL, BD Biosciences, Shanghai, China). 0.1 mL of cell suspension (2×10^7^ cells/ml) were injected subcutaneously at one site on each flank of female nude mice. Mice were then injected subcutaneously daily for 32 days with vehicle, androstenedione (Δ4A, 100μg/d), or androstenedione plus letrozole (10μg/d) from the day of inoculation. Tumor volume changes were recorded every 3 days by two perpendicular diameter measurements [34].

### Quantitative RT-PCR (RT-qPCR)

Total RNA was isolated using MagMAX™ mirVana™ Total RNA Isolation Kit (Thermo Fisher Scientific, Waltham, MA, USA). Reverse transcription of miRNA and other targets was carried out using miRNA-specific primers (3’-TGGCTCAGTTCAGCAGGAACAG-5’) and a PrimeScript RT Reagent Kit (Takara, Dalian, China), respectively. Expression levels of miR-1271 and various targets were then assayed using All-in-One™ miRNA RT-qPCR Reagent Kit (GeneCopoeia, Guangzhou, China) according to the manufacturer’s protocol, with *18S* and *U6* being used as internal controls for different targets and miR-1271, respectively. The primer sequences used were listed in Supplementary Table 2. Relative expression levels were determined using 2^–ΔΔCT^ method [35].

### Immunoblotting

Immunoblotting was performed as described elsewhere [36]. Membranes were then incubated with various primary antibodies (Supplementary Table 3) in blocking solution overnight at 4°C. Final signals were detected by using an enhance chemiluminescence kit (Amersham Biosciences, Shanghai, China), with the assistance of an automated western blot processors (Clinx Science, Shanghai, China). Densitometric analysis was performed by using Image J software and normalized for β-ACTIN staining [37].

### Luciferase assay

pLightSwitch-Luc-*DDIT3*/3’UTR, pLightSwitch-Luc-*ESR1* and blank vectors were purchased from SwitchGear Genomics (Shanghai, China). pLightSwitch-Luc-*DDIT3*/3’UTR is a luciferase reporter with the 3’UTR of *DDIT3* and pLightSwitch-Luc-*ESR1* is a luciferase reporter with the promoter region of *ESR1* gene. The site-directed mutagenesis of the miR-1271 binding-site in *DDIT3*/3’UTR was achieved using QuikChange Site-Directed Mutagenesis Kit (Agilent, Santa Clara, CA, USA). For luciferase assay, 2 d after transfection either with miR-1271 mimics or mimic controls, or with pLenti-GIII-CMV-DDIT3 or pLenti-GIII-CMV vectors, COS-1 cells were incubated with 100 ng reporter plasmids using FuGENE® HD Transfection Reagent (Promega, Beijing, China) for 48 h, followed by measurement of luciferase activity using LightSwitch Assay Reagents according to the manufacturer’s instructions. COS-1 cells were chosen for their high efficiency of transfection and for the relative low level of endogenous miR-1271 expression (data not shown).

### Chromatin immunoprecipitations (ChIP)

MCF7 cells transfected with pLenti-GIII-CMV-DDIT3 or with pLenti-GIII-CMV vectors were treated with letrozole (10^-5^mol/l) for 2 days, followed by ChIP assay as described elsewhere [38]. Recovery and preparation of DNA was followed by PCR using primers flanking the three regions of *ESR1* promoter. The primers used for ChIP assay are listed in the Supplementary Table 2. To confirm the binding site of DDIT3 on *ESR1* promoter, human ESR1 promoter was cloned into pCI Mammalian Expression Vector (Promega, Madison, WI, USA; designated as pCI-*ESR1*-promoter). The site-directed mutagenesis of the DDIT3 binding-site in *ESR1* promoter was achieved using QuikChange Site-Directed Mutagenesis Kit (Agilent, Santa Clara, CA, USA; designated as pCI-mu-*ESR1*-promoter). For the subsequent ChIP assay, pCI-*ESR1*-promoter or pCI-mu-*ESR1*-promoter were co-transfected with pCMV-DDK-tagged-DDIT3 (OriGene Technologies, Beijing, China) into HeLa cells. This cell type was chosen because it has been shown in the Human Protein Atlas database that HeLa cells possesses negligible expression level of endogenous *ESR1*. 48 h later, transfected cells were treated with letrozole (10^-5^mol/l) or control (DMSO) for another 24 h, followed by ChIP assay using P3 primers as described above.

### Statistical analysis

Quantitative data, presented as mean ± S.E.M., were analyzed for statistically significant differences using ANOVA with post hoc tests (Tukey test) wherever appropriate. The correlation between miR-1271 expression and *DDIT3* mRNA level was assessed using the *Pearson Chi-Square* test. *P*<0.05 was considered significant.

## SUPPLEMENTARY MATERIALS FIGURES AND TABLES


