# Supplementary material for: miR-1271 inhibits ERα expression and confers letrozole resistance in breast cancer
Source: Oncotarget. 2017 Nov 9;8(63):107134–48. doi: 10.18632/oncotarget.22359 (PMC5739802; doi:10.18632/oncotarget.22359)
Supplement: Supplementary file 1 [file oncotarget-08-107134-s001.pdf]

miR-1271 inhibits ERα expression and confers letrozole resistance in breast cancer

SUPPLEMENTARY MATERIALS

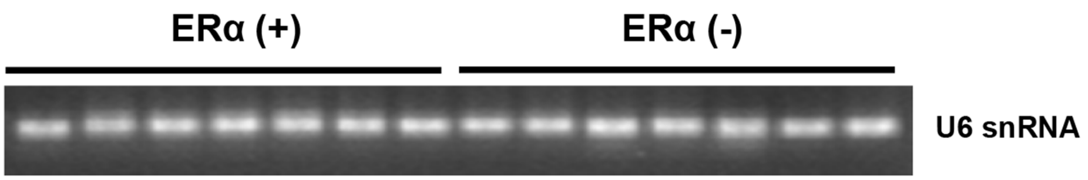

Supplementary Figure 1: Detection of U6 snRNA in 7 pairs of BCa specimens, as presented in Figure 1A, was achieved using RT-PCR.

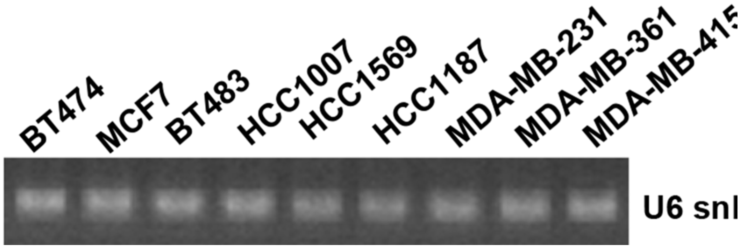

Supplementary Figure 2: Detection of U6 snRNA in different BCa cells, as presented in Figure 1D, was assessed using RT-PCR.

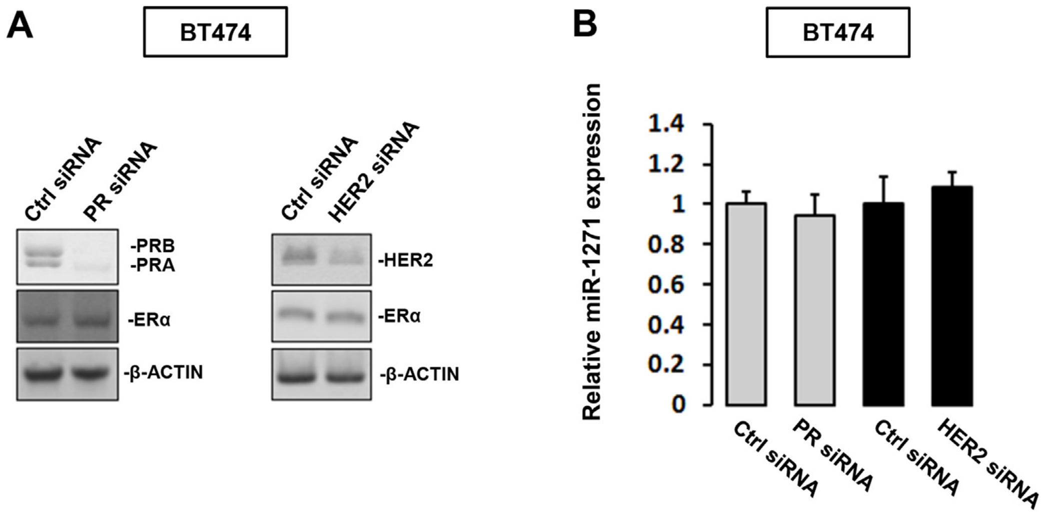

Supplementary Figure 3: Effect of PR or HER2 status on miR-1271 expression levels. **(A)** BT474 cells were transiently transfected with PR siRNA, HER2 siRNA and their corresponding negative controls (Ctrl siRNA), respectively. 48 h later, BT474 cells were harvested and subjected to immunoblotting analysis of ERα expression. **(B)** BT474 cells were transiently transfected with PR siRNA, HER2 siRNA and their corresponding negative controls (Ctrl siRNA), respectively. 48 h later, BT474 cells were harvested and subjected to RT-qPCR analysis of mir-1271 expression.

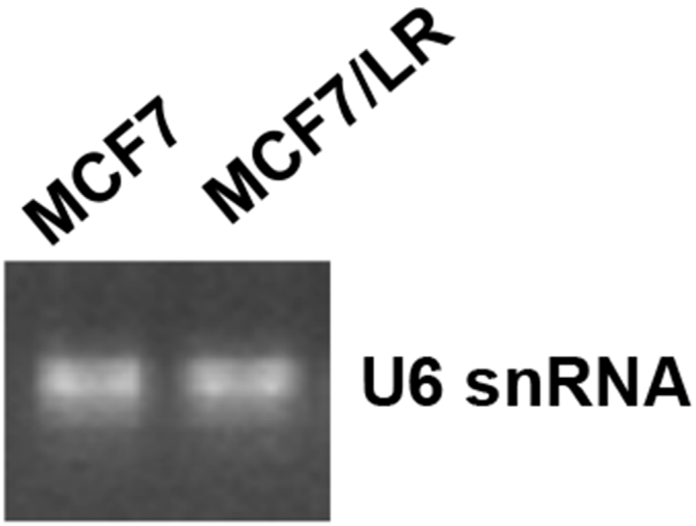

Supplementary Figure 4: Detection of U6 snRNA in different BCa cells, as presented in Figure 1F, was assessed using RT-PCR.

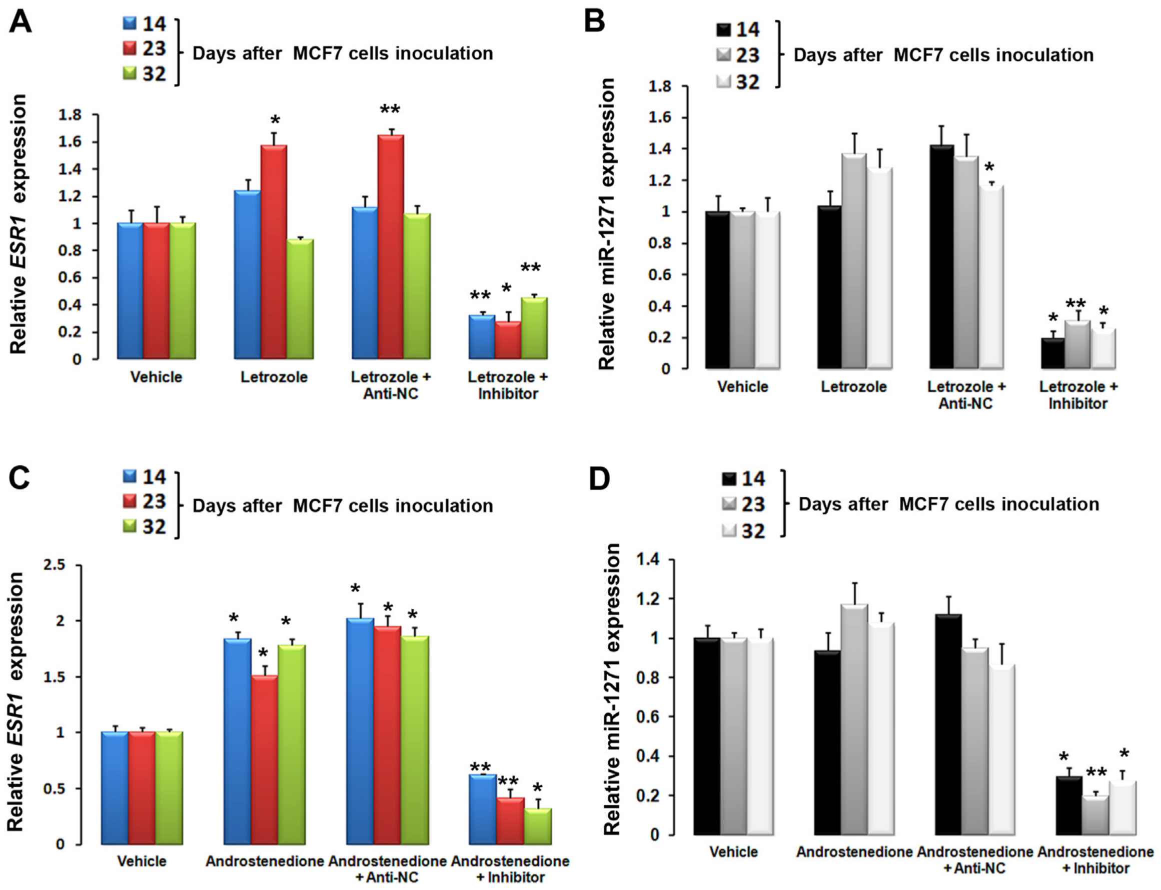

Supplementary Figure 5: Effects of letrozole treatment or androstenedione treatment on ESR1 and miR-1271 expression in MCF7 tumor xenografts. Letrozole or androstenedione-treated xenografts were collected at 14, 23 and 32 days after MCF7 cells inoculation, analyzed by RT-qPCR, and were compared with vehicle-treated xenografts collected at different timepoints (control). Letrozole treatment stimulated ESR1 expression at D 23, but exerted no effects at D 32. In contrast, androstenedione treatment constantly stimulated ESR1 expression at D 23 and D 32. More importantly, regardless of letrozole treatment or androstenedione treatment,, we constantly observed a significant decrease in both ESR1 and miR-1271 expression levels in the MCF7 cells transfected with miR-1271 inhibitors. These results confirm that (1) by using miR-1271 inhibitors treatment, we could suppress miR-1271 expression levels for a relatively long period of time (about 35 days); (2) miR-1271 expression appeared to be positively correlated to ESR1 expression in BCa cells. $^{*}P<0.05$ or $^{**}P<0.01$ when compared to the values observed in vehicle-treated xenografts at different timepoints.

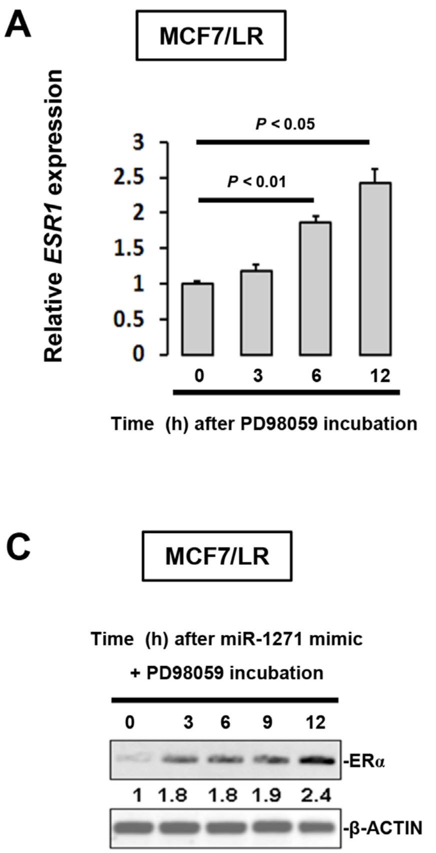

Supplementary Figure 6: Effect of combined treatment with miR-1271 mimic and PD98059 on *ESR1* or ERα expression levels. (A) MCF7/LR cells were incubated with the MAPK pathway inhibitor PD98059 (10 μM) for various durations, followed by RT-qPCR analysis of *ESR1* expression. **(B)** MCF7 and MCF7/LR cells were incubated with PD98059 (10 μM) for 6 h, followed by RT-qPCR analysis of *ESR1* expression. **(C)** MCF7/LR cells were incubated with miR-1271 mimic and PD98059 for different durations, followed by immunoblotting analysis of ERα expression.

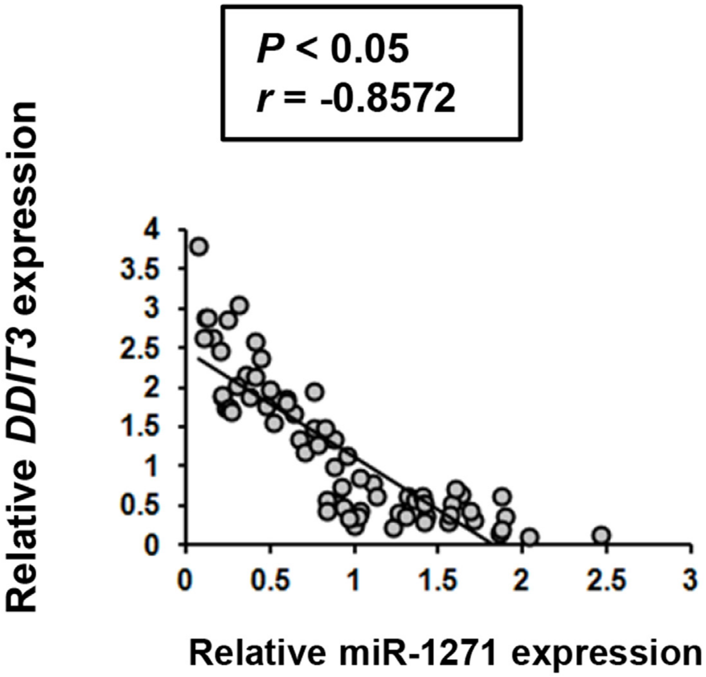

Supplementary Figure 7: RT-qPCR analysis along with *Pearson Chi-Square* test revealed that *DDIT3* mRNA level was inversely correlated with miR-1271 level in clinical BCa samples.

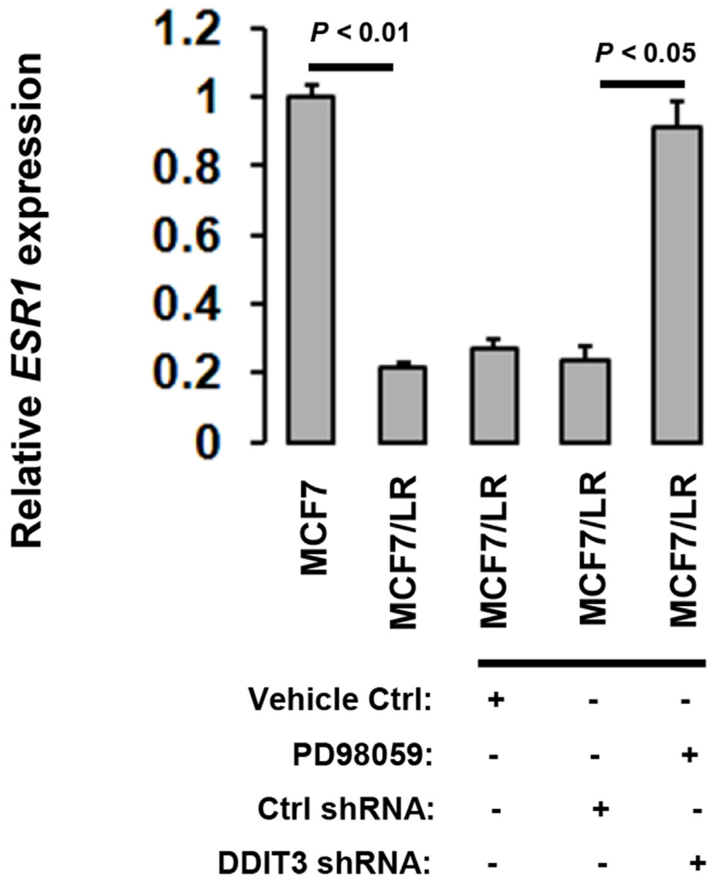

Supplementary Figure 8: Effect of combined treatment with DDIT3 shRNA and PD98059 on *ESR1* expression levels in MCF7/LR cells. MCF7/LR cells were transfected with DDIT3 shRNA or shRNA NC for 48 h, followed by incubation with PD98059 for another 6 h. Subsequently, the relative expression levels of *ESR1* mRNA in different experimental groups were determined using RT-qPCR.

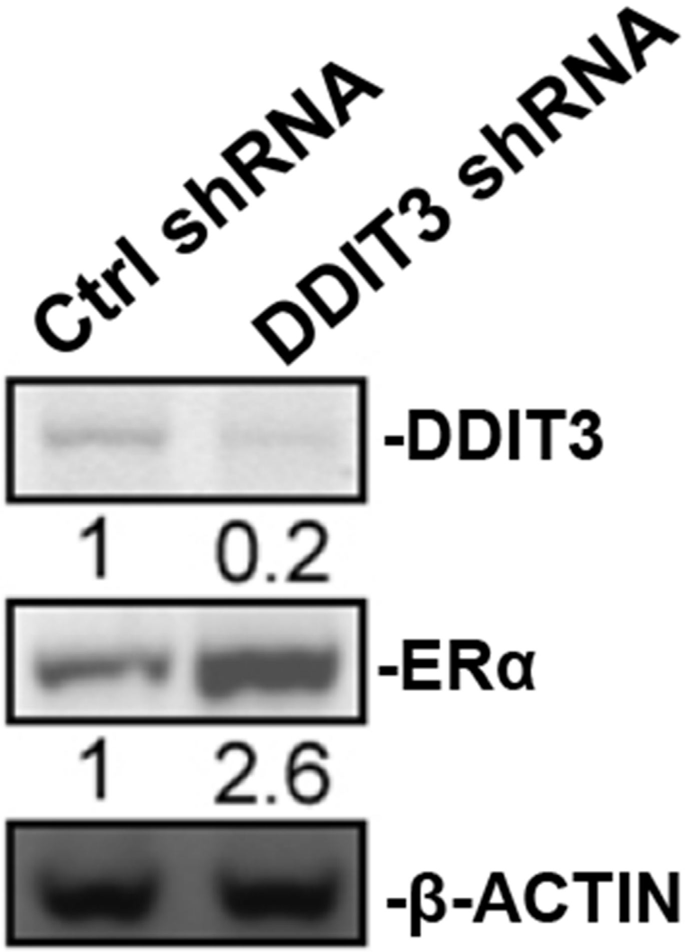

Supplementary Figure 9: Establishment of MCF7 cells stably deficient of DDIT3 expression was confirmed using immunoblotting analysis. Ablation of endogenous DDIT3 expression significantly stimulated ERα expression levels.

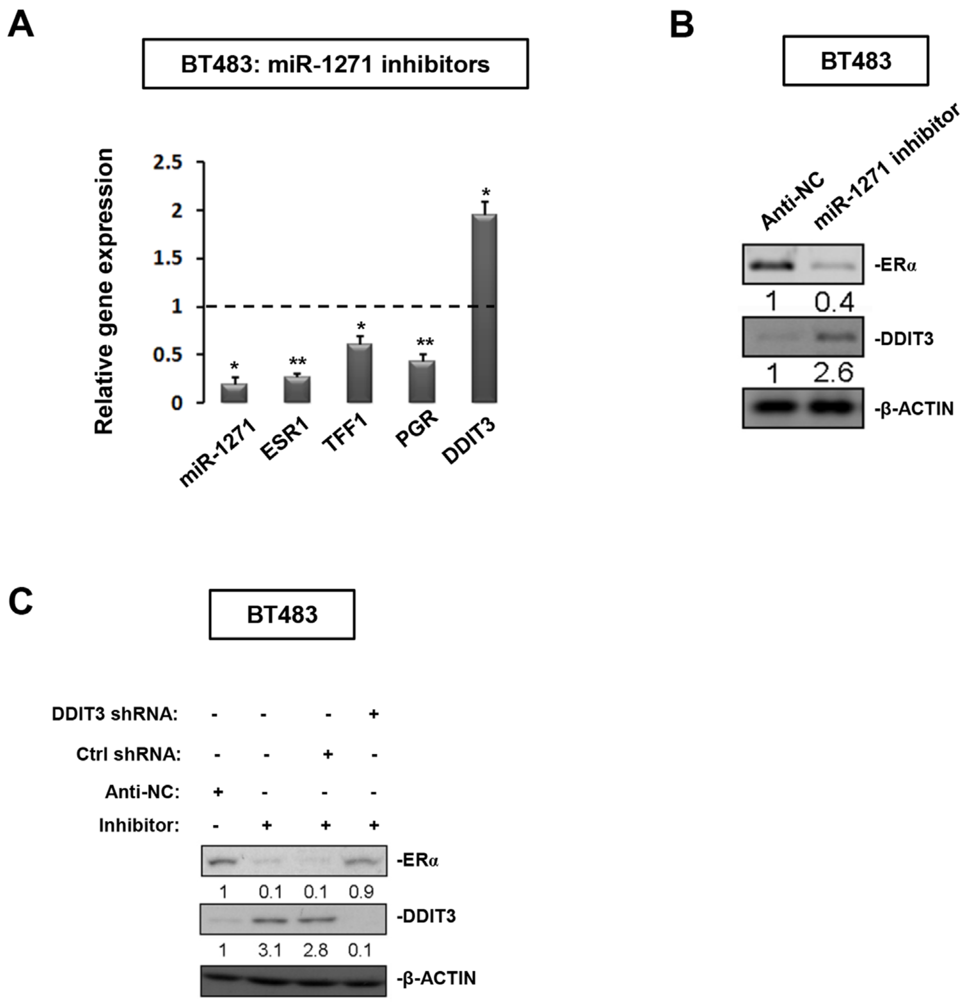

Supplementary Figure 10: Verification of the effect of miR-1271/DDIT3/ERα cascade in another BCa luminal cell line (BT483 cells). We transiently transfected BT483 cells with miR-1271 inhibitors or with Anti-NC as described above. 48 h later, cells were subjected to RT-qPCR **(A)** and immunoblotting analyses **(B)** to reveal the expression levels of ERα pathway and DDIT3. **(C)** Repression of DDIT3 expression level using shRNA treatment could effectively rescue the ERα expression levels even in the presence of miR-1271 inhibitors.

Supplementary Table 1: Characteristics of the 70 patients with primary breast cancer from Department of Breast Surgery in Liaoning Cancer Hospital & Institute (Shenyang, China)

	Number of patients (70)
**Age (years)**	
**≤ 50**	31 (44.3%)
**> 50**	39 (55.7%)
**Macroscopic tumor size**	
**≤ 30 mm**	25 (35.7%)
**> 30 mm**	45 (64.3%)
**Lymph node status**	
**≤ 3 involved**	41 (58.6%)
**> 3 involved**	29 (41.4%)
**Scarff-Bloom-Richardson classification**	
**1**	10 (14.3%)
**2**	17 (24.3%)
**3**	43 (61.4%)
**Estrogen Receptor status**	
**Positive**	43 (61.4%)
**Negative**	27 (38.6%)
**Progesterone Receptor status**	
**Positive**	37 (52.9%)
**Negative**	33 (47.1%)
**HER2 status**	
**Positive**	26 (37.1%)
**Negative**	44 (62.9%)

Supplementary Table 2: The primer sequences used in the current study

Targets	GenBank access NO. or references	Primer sequence	Product size	Annealing Temperature	Concentration		Cycle
					cDNA	Primer	
miR-1271	Yang WM, *et al*. 2016	F:TTGGCACCTAGCAAGCACTCA	90	55	2 ng/μl	0.5 μM	40
		R: miScript universal primer (Qiagen)					
U6		F: CTCGCTTCGGCAGCACA	94				
		R: AACGCTTCACGAATTTGCGT					
ESR1	NM_000125.3	F: GTCGCCTCTAACCTCGGG	131	60			
		R: GCTTTGGTGTGGAGGGTCAT					
TFF1	NM_003225.2	F: ATACCATCGACGTCCCTCCA	106	58			
		R: TGGGACTAATCACCGTGCTG					
PGR	NM_001202474.3	F: TCCTCATAGGCCCAGCTCTT	117	57			
		R: TTAGTGTGGTGGCAGCTGAG					
DDIT3	NM_001195053.1	F: GACCTGCAAGAGGTCCTGTC	124	58			
		R: CAGTCAGCCAAGCCAGAGAA					
18S	Li W, *et al*. 2011	F: CTCGCCGCGCTCTACCTACCTA	120	60			
		R: ATGAGCCATTCGCAGTTTCACTGTA					
P1	NM_000125.3	F: CCAGGGCTTAAAGGTTAGGA	91	60			
		R: AAAATAGCACCCAACTGCTTCAG					
P2	NM_000125.3	F: GTCCTTCACATTTCTTTTCCTT	142				
		R: GTATCACGAGGGGAGTGAGCTTT					
P3	NM_000125.3	F: -CATGACGCTGTGCCAGGGAG	123				
		R: TGTAACCATGAATCCCTTGT					

1. Yang WM, Min KH, Lee W. MiR-1271 upregulated by saturated fatty acid palmitate provokes impaired insulin signaling by repressing INSR and IRS-1 expression in HepG2 cells. *Biochem Biophys Res Commun*. 2016; 478: 1786-1791.
2. Li W, Wu ZQ, Zhao J, Guo SJ, Li Z, Feng X, Ma L, Zhang JS, Liu XP, Zhang YQ. Transient protection from heat-stress induced apoptotic stimulation by metastasis-associated protein 1 in pachytene spermatocytes. *PLoS One*. 2011; 6: e26013.

Supplementary Table 3: Sources of antibodies and the working dilutions that were used in the current study

Antibody	Vendor	Catalog no.	Dilutions
Rabbit anti-ERα	CST (Shanghai, China)	#8644	1:1000
Rabbit anti-ERBB-2	CST (Shanghai, China)	#2165	1:1000
Rabbit anti-GRB2	CST (Shanghai, China)	#3972	1:1200
Rabbit anti-p-MAPK	CST (Shanghai, China)	#4370	1:1000
Rabbit anti-MAPK	Santa Cruz Biotechnology (Shanghai, China)	sc-292838	1:2000
Rabbit anti-p-p90RSK	CST (Shanghai, China)	#9341	1:1500
Rabbit anti-p90RSK	GeneScript (Nanjing, China)	A00603-100	1:1000
Rabbit anti-β-ACTIN	Thermo Fisher Scientific (Shanghai, China)	PA1-46296	1:3000
Mouse anti-DDIT3	Abcam, (Hangzhou, China)	ab11419	1:500 (IB) 2μg/reaction (IP)
Goat anti-Rabbit IgG-HRP secondary antibody	Abcam, (Hangzhou, China)	ab97200	1:10000
Goat anti-Mouse IgG-HRP secondary antibody	Santa Cruz Biotechnology (Shanghai, China)	sc-2005	1:2500
